# A New PETase from the Human Saliva Metagenome and Its Functional Modification via Genetic Code Expansion in Bacteria

**DOI:** 10.1002/anie.202216963

**Published:** 2023-01-24

**Authors:** Mirren F. M. White, Stephen Wallace

**Affiliations:** ^1^ Institute of Quantitative Biology Biochemistry and Biotechnology School of Biological Sciences University of Edinburgh, King's Buildings Alexander Crum Brown Road Edinburgh EH9 3FF UK

**Keywords:** Biocatalysis, Degradation, Genetic Code Expansion, Hydrolases, Plastics

## Abstract

The discovery and engineering of new plastic degrading enzymes is an important challenge in chemical biotechnology to enable transition to a more sustainable and circular plastics economy. This field has so far yielded a range of enzymes and microbial pathways for the recycling and valorization of plastic waste. New research from Uttamapinant et al. reports the discovery of a novel polyethylene terephthalate (PET) hydrolase from the human saliva metagenome that displays improved properties and catalytic performance over previously characterized PET hydrolases (PETases). The authors also demonstrate the site‐specific incorporation of a photocaged unnatural amino acid, 2,3‐diaminopropionic acid (DAP), which upon photodecaging enables covalent binding of DAP to the PET surface. Thus, this work highlights metagenomic datasets as an untapped source of new PET degrading enzymes and the chemical modification of PETases via genetic code expansion, enabling new biotechnologies for the circular plastics economy.

## Graphical abstract

Uttamapinant et al. reported the discovery of new polyethylene terephthalate (PET) degrading enzymes from aquatic and human saliva metagenomes. Their catalytic performance is up to 83 times higher than known PETases. Site‐specific incorporation of an unnatural amino acid results in a stable acyl‐enzyme intermediate and a PET‐enzyme conjugate which can be imaged via confocal microscopy.

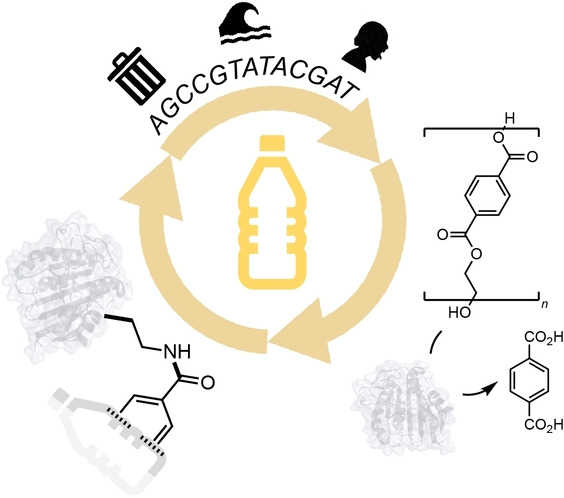

Plastic is a ubiquitous material across modern‐day society. However, the mismanagement of plastic waste has led to the accumulation of this synthetic material in ecosystems worldwide, resulting in reduced biodiversity and widespread plastic pollution. Polyethylene terephthalate (PET) is the major constituent of single‐use plastics and accounts for the majority of global plastic waste. In 2016, scientists sampling the environment surrounding a PET recycling facility in Japan isolated a novel bacterium, *Ideonella sakaiensis*, that had evolved the metabolic chemistry to both breakdown and assimilate PET as a sole carbon and energy source.^[1]^ This led to the discovery of a novel enzyme, *Is*PETase, that could catalyze the hydrolysis of PET into its constituent monomers, ethylene glycol and terephthalic acid (TPA). As a result, this sparked a series of protein engineering and directed evolution endeavors to increase the activity of *Is*PETase and other PET hydrolases (PETase) for industrial use.[^2^, ^3^, ^4^, ^5^, ^6^, ^7^, ^8^] In parallel, this early work also led to increased efforts to identify new plastic‐degrading enzymes from metagenomic datasets isolated from diverse plastic‐contaminated environments.[^9^, ^10^, ^11^] In this recent report by Uttamapinant et al.,^[12]^ the authors identified new PETase enzymes from marine and human saliva metagenomes. The catalytic activities of these enzymes are higher than many native and engineered PETase enzymes reported to date, and therefore represent attractive targets for future protein engineering, directed evolution and industrial biotechnology. This study also demonstrates the incorporation of a photocaged 2,3‐diaminopropionic acid (DAP)‐containing unnatural amino acid into the active site of PETase MG8 via AMBER codon suppression, transforming the enzyme upon UV irradiation into a covalent PET binder.

The authors began by searching the open‐access MGnify metagenomic database for non‐redundant protein homologs containing the Ser/Asp/His catalytic triad of *Is*PETase. This generated 629 amino acid sequences from close to 200 000 samples isolated from human or aquatic environments. Candidate sequences were narrowed based on clustering to known highly active PETases within a sequence similarity network. Shortlisted sequences were manually curated based on the nature of the isolation environment—e.g. selecting for high salinity and marine environments located near PET‐containing “garbage patches” (Figure 1a). This generated 10 candidate sequences that were distinct from known PETases for further investigation—MG1‐MG7 and MG8‐MG10, from marine and human metagenomes, respectively. Using BLAST tool, all candidates were putatively assigned to be of bacterial origin—MG1‐MG8 from Gram‐negative *Pseudomonadota*, and MG9‐MG10 from Gram‐positive *Actinomycetota*. All candidates were expressed in *Escherichia coli*, isolated, and refolded *in vitro* from insoluble inclusion bodies for characterization of their PET degrading activity. Intriguingly, all except MG4 also contained putative secretion signal tags, indicating their potential role in the degradation of extracellular ester substrates in native environments. All of the candidate enzymes displayed NaCl‐dependent activity (up to 5 M NaCl), and MG1, MG7, MG8 and MG10 displayed the highest activity toward the PET‐derived substrate *bis*(2‐hydroxyethyl) terephthalate (BHET). Following a subsequent assay using PET powder, the authors narrowed their focus to MG8, a hydrolase enzyme isolated from a human saliva metagenome sample. Overall, MG8 displayed the highest activity across multiple tests, and at 55 °C the enzyme produced ca. 5–83 fold increased TPA from PET when compared to *Is*PETase, DuraPETase and ThermoPETase (Figure 1b). The authors also identified an unusual structural feature in MG8 to which they attribute this increased activity—a three‐residue extension of the catalytic loop within the enzyme active site containing a phenylalanine at position 250 that supposedly has evolved to increase binding to the hydrophobic PET surface. MG8 is the first example of a PETase isolated from the human saliva metagenome, displaying improved catalytic properties and intriguing additional structural motifs that make it a notable addition to the growing family of plastic‐degrading enzymes. Improving the activity and productivity of MG8 towards industrial and post‐consumer PET samples will be essential for the application of MG8 at scale for PET recycling. Additionally, the high cost of enzymes containing unnatural amino acids should be addressed as this is currently prohibitive at scale.

**Figure 1:**
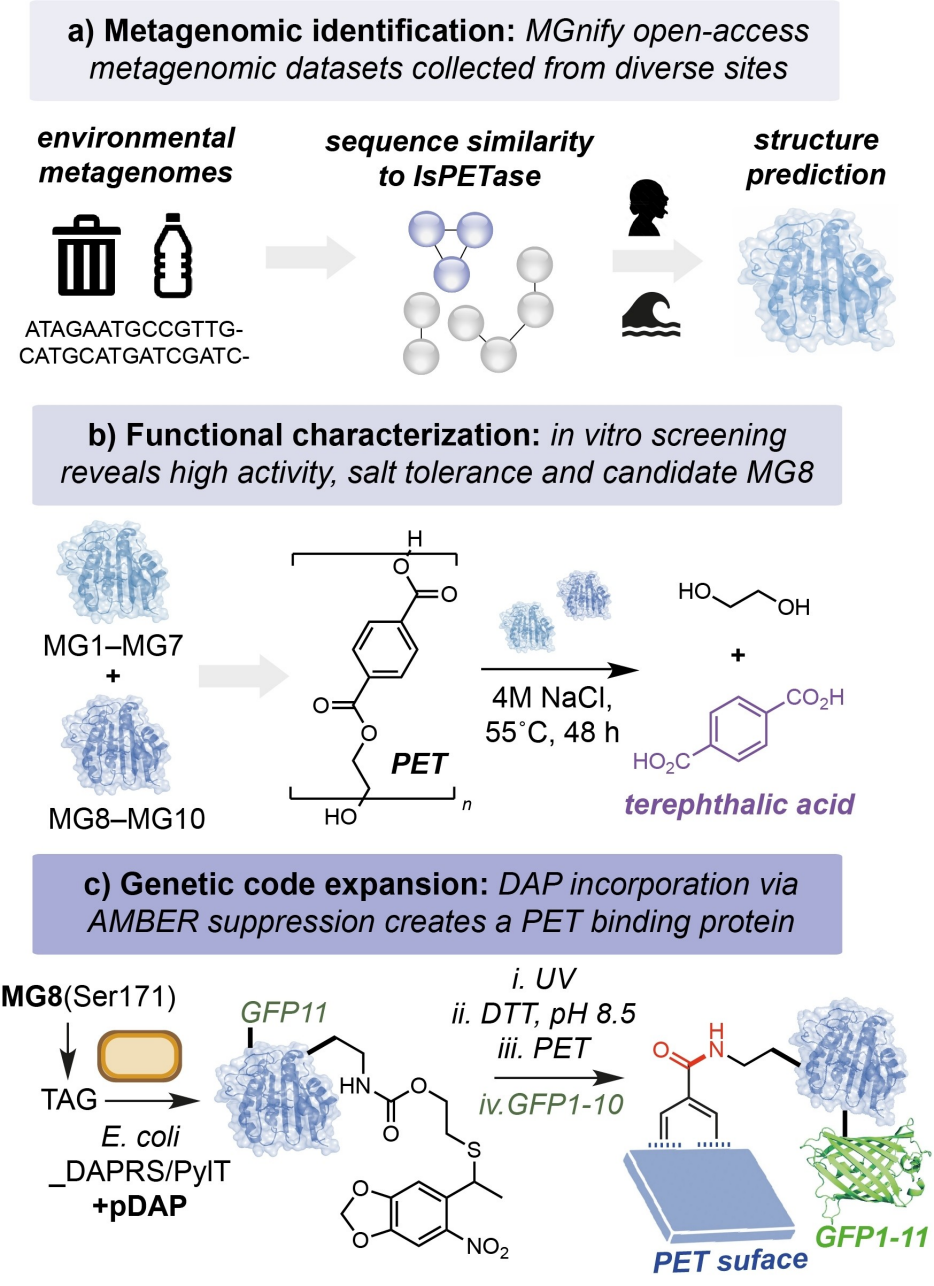
A) Metagenomic database search for novel PETase enzymes. B) Characterization of candidates MG1‐10 identified from aquatic and human saliva metagenomes. C) Genetic incorporation of an unnatural amino acid via AMBER codon suppression to produce a PET binding protein.

Following characterization of the novel PETases, the authors focused on the modification of MG8 as a tool for the functional display of proteins on the surface of PET. This approach offers a novel method to co‐localize enzymes to plastic surfaces for enhanced biodegradation/upcycling, or as a platform for the development of wearable biosensors using waste PET. The authors hypothesized that by incorporating the non‐canonical amino acid DAP in place of the catalytic serine residue in the enzyme active site, the acyl‐enzyme intermediate that is formed during the breakdown of PET would be instead stabilized covalently, resulting in binding of the enzyme to the plastic surface. This elegant approach also addresses a critical challenge in the field of PETase‐directed evolution: overcoming the native catalytic properties of residues surrounding the oxyanion hole to catalyze both formation *and* hydrolysis of the acyl‐enzyme is extremely difficult. Here, site‐specific modification of S171 with a photocaged DAP (pDAP) via genetic code expansion would generate MG8^S171DAP^ upon photodecaging under basic conditions, which would in turn form a stable amide intermediate with PET during the catalytic cycle. To this end, co‐expression of MG8^S171TAG^ alongside the orthogonal translation machinery pDAPRS/PyltRNA_CUA_ in *E. coli* generated MG8^S171pDAP^ that could be quantitatively converted to MG8^S171DAP^ by UV light (365 nm) and incubation in the presence of DTT at pH 8.5. The authors then proposed using protein fusions with DAP‐modified MG8 to display a variety of functional handles for bioconjugation. They demonstrate this using a split‐GFP system to enable the stable attachment and visualization of GFP on the surface of PET and other bio‐based and biodegradable plastics, including polybutylene succinate, polycaprolactone and polylactic acid (Figure 1c).

The enzyme‐catalyzed degradation and recycling of PET waste is a critical pillar for the future sustainable manufacturing of plastic materials, in addition to the use of plastic as a feedstock for the circular bioeconomy via microbial upcycling.^[13]^ This study by Uttamapinant et al. demonstrates the exploration of open‐access metagenomic datasets to uncover new PET‐degrading enzymes and reports the discovery of one of the most active PETases in the literature to date. MG8 is also the first PETase to be isolated from a human saliva metagenome, indicating that the extensive use of plastic materials and microplastics in food has triggered the evolution of PETases in the human oral cavity. Using genetic code expansion, the authors demonstrate the transformation of MG8 PETase into a PET‐binding enzyme through site‐specific modification of the active site with a reactive unnatural amino acid, paving the way for the functional modification of PET and the study of enzyme binding to diverse plastic surfaces. Together, this work demonstrates the remarkable metabolic plasticity of microorganisms to utilize diverse “waste” carbon feedstocks, and how the bioinformatic exploration of metagenomic datasets collected from diverse microbial environments can be combined with modern synthetic biology tools to generate new biotechnologies for the circular chemical economy.

## Conflict of interest

The authors declare no conflict of interest.

## Data Availability

The data that support the findings of this study are available from the corresponding author upon reasonable request.
